# Characterization of a New Thermostable and Organic Solution-Tolerant Lipase from *Pseudomonas fluorescens* and Its Application in the Enrichment of Polyunsaturated Fatty Acids

**DOI:** 10.3390/ijms24108924

**Published:** 2023-05-18

**Authors:** Zhiming Hu, Liangcheng Jiao, Xiaoman Xie, Li Xu, Jinyong Yan, Min Yang, Yunjun Yan

**Affiliations:** Key Laboratory of Molecular Biophysics of the Ministry of Education, College of Life Science and Technology, Huazhong University of Science and Technology, Wuhan 430074, China

**Keywords:** *Pseudomonas fluorescens*, lipase, characterization, hydrolysis, polyunsaturated fatty acids

## Abstract

The search for and characterization of new lipases with excellent properties has always been urgent and is of great importance to meet industrial needs. In this study, a new lipase, *lipB,* from *Pseudomonas fluorescens* SBW25, belonging to the lipase subfamily I.3, was cloned and expressed in *Bacillus subtilis* WB800N. Enzymatic properties studies of recombinant LipB found that it exhibited the highest activity towards *p*-nitrophenyl caprylate at 40 °C and pH 8.0, retaining 73% of its original activity after incubation at 70 °C for 6 h. In addition, Ca^2+^, Mg^2+^, and Ba^2+^ strongly enhanced the activity of LipB, while Cu^2+^, Zn^2+^, Mn^2+^, and CTAB showed an inhibiting effect. The LipB also displayed noticeable tolerance to organic solvents, especially acetonitrile, isopropanol, acetone, and DMSO. Moreover, LipB was applied to the enrichment of polyunsaturated fatty acids from fish oil. After hydrolyzing for 24 h, it could increase the contents of polyunsaturated fatty acids from 43.16% to 72.18%, consisting of 5.75% eicosapentaenoic acid, 19.57% docosapentaenoic acid, and 46.86% docosahexaenoic acid, respectively. The properties of LipB render it great potential in industrial applications, especially in health food production.

## 1. Introduction

As one of the most important lipolytic enzymes, lipases (triacylglycerol acyl hydrolases, EC 3.1.1.3) possess the natural ability to catalyze hydrolysis, esterification, interesterification, and transesterification reactions in aqueous or non-aqueous media, exhibiting high levels of regio-, chemo-, and enantioselectivity [1,2,3]. Lipases play a crucial role in multiple industrial fields, such as food, pharmaceuticals, cosmetics, detergents, agrochemicals, leather, textiles, and paper. Compared to plant- and animal-derived lipases, microbial lipases are more attractive for application due to their large-scale production and genetic manipulation capabilities [4,5].

Among microbial lipases, bacterial lipases, especially those derived from *Pseudomonas* and *Burkholderia*, such as the lipases from *Pseudomonas fluorescens* [6], *Pseudomonas brenneri* [5], and *Burkholderia ubonensis* [7], exhibit higher thermal stability and tolerance to extreme physicochemical parameters than fungal lipases [8]. So far, several notable contributions have been made toward the screening, purification, and characterization of bacterial lipases [9,10]. However, with the rapid development of modern biochemical industries, more lipases with excellent properties are needed to meet the growing demand for industrial enzymes. Consequently, the investigation of new lipases with unique characteristics (such as high activity, thermal stability, organic solvent resistance, etc.) is still urgent and of vital importance. LipB, a predicted lipase produced from *P. fluorescens* SBW25, may have outstanding characteristics but has not yet been explored.

As known, n-3 polyunsaturated fatty acids (PUFAs) such as eicosapentaenoic acid (EPA), docosapentaenoic acid (DPA), and docosahexaenoic acid (DHA) are long-chain polyunsaturated fatty acids, which are essential for human health and play an important role in decreasing the risk of diseases such as diabetes, neurological, inflammatory, and cardiovascular [11,12]. The performance of n-3 PUFAs is greatly influenced by their content and form. Compared to fatty acid ethyl esters (FAEEs) and free fatty acids (FFAs), n-3 PUFAs existing in the form of glycerides possess higher oxidative stability and bioavailability [13]. Since the human body lacks the enzymes required for n-3 PUFA synthesis, n-3 PUFAs can only be obtained through diet [14,15]. Unfortunately, the amount of n-3 PUFAs in the diet is very low and cannot meet people’s requirements, resulting in an increasing demand for nutraceuticals containing high concentrations of n-3 PUFAs [16].

Until now, chemical methods such as urea complexation, distillation, low-temperature crystallization, and supercritical fluid extraction have been commonly used to enrich n-3 PUFAs in industry, which is usually accompanied by obvious disadvantages, for instance, the existence of FAEEs or FFAs, high energy consumption, poor quality, and low yield [17,18]. In contrast, enzymatic methods using lipases as the catalyst to enrich n-3 PUFAs in the form of glycerides by hydrolyzing saturated fatty acids (SFAs) and monounsaturated fatty acids (MUFAs) display considerable advantages due to lower energy expenditure and minimal waste production [19]. Several new microbial lipases have been characterized and employed to enrich PUFAs, such as *Geotrichum candidum* lipase [20] and *Trichosporon* sp. F1-2 lipase [21]. Additionally, in many reports, *Candida rugosa* lipase, *Candida antarctica* lipase, and *Thermomyces lanuginosa* lipase have been used for enrichment studies in fish oil, with the best result being a twofold increase in PUFA content [12,22,23]. Many lipases used in enzymatic methods possess low hydrolytic activity, poor enrichment capacity, and lack fatty acid selectivity [12,21,24]. Therefore, it is important to screen novel lipases that can efficiently enrich n-3 PUFAs.

In this study, *lipB,* a previously predicted potential lipase gene of *P. fluorescens* SBW25, was synthesized after codon optimization and successfully expressed in *Bacillus subtilis* WB800N. The recombinant LipB was characterized in detail and then employed to enrich polyunsaturated fatty acids in fish oil, seeking a lipase capable of ameliorating the aforementioned problems.

## 2. Results and Discussion

### 2.1. Sequence Analysis and Structural Homology Modeling

**Figure 1:**
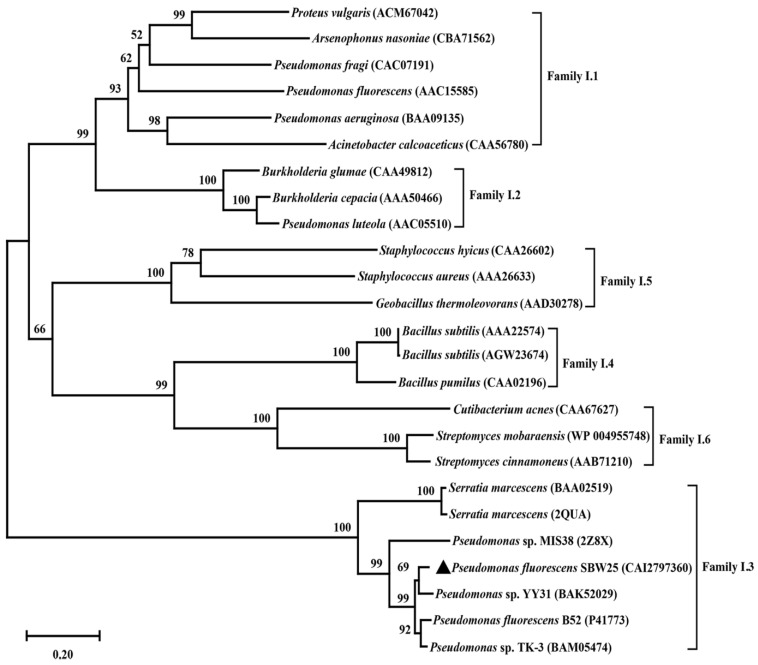
Phylogenetic analysis of LipB. All the lipases shown belong to the bacterial lipolytic enzyme family I. The source strain, *Pseudomonas fluorescens* SBW25, of the target lipase was marked with black triangles.

**Figure 2:**
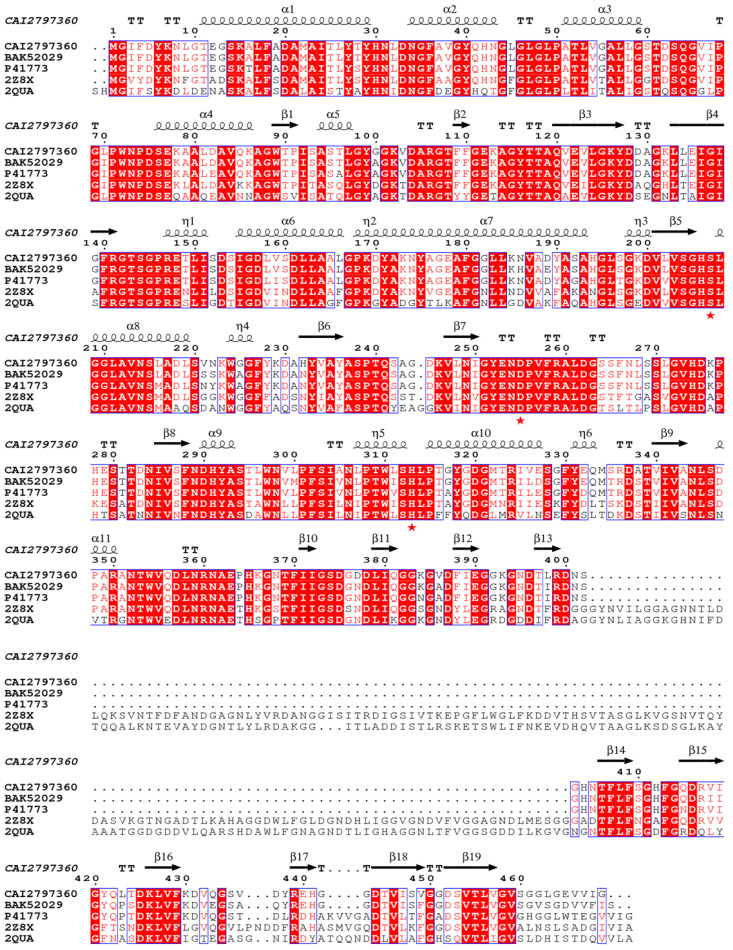
Multiple sequence alignments of LipB and other lipolytic enzymes belonging to the subfamily I.3. LipB was aligned with *Pseudomonas* sp. YY31 lipase (BAK52029), *P. fluorescens* B52 lipase (P41773), *Pseudomonas* sp. MIS38 lipase (2Z8X), and *Serratia marcescens* lipase (2QUA) using the programs ClustalW and ESpript. The catalytic triad (Ser207, Asp255, and His313) was emphasized with red stars (★).

**Figure 3:**
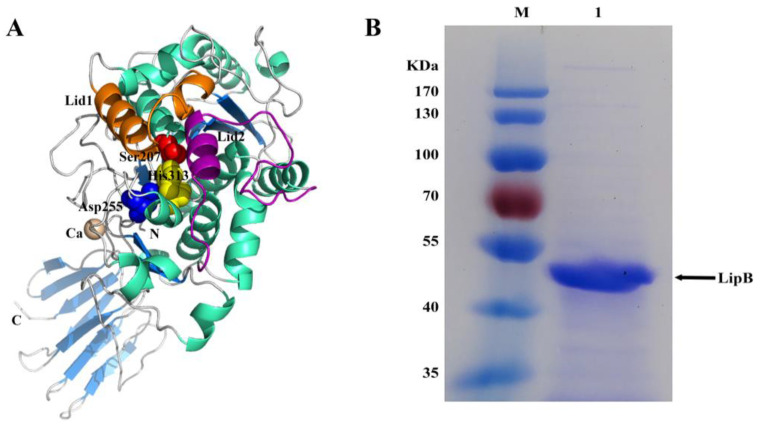
A 3D model and the purification of LipB. (**A**) The stereo view of the 3D model of LipB. The catalytic triad (Ser207, Asp255, and His313) was indicated as red, blue, and yellow spheres, respectively. The α-helix, β-sheet, and random coil were colored in cyan, marine, and gray, respectively. The calcium ion was shown as a wheat sphere. The putative lid1 and lid2 helices were colored magenta and orange, respectively. (**B**) SDS-PAGE analysis of purified recombinant LipB. Lane M, protein mass marker (Fermentas, SM0671). Lane 1, purified recombinant LipB.

The open reading frame of *lipB* was predicted to encode a lipase, but it had not been experimentally demonstrated. To verify the prediction, a bioinformatics analysis of the *lipB* gene was first performed. It was found that *lipB* had a total length of 1410 bp and encoded a protein consisting of 470 amino acids with a predicted molecular weight of 49.36 kDa and an isoelectric point of 5.25. BLASTP analysis revealed that LipB exhibited an amino acid identity of 93% to *Pseudomonas* sp. YY31 lipase (LipYY31; BAK52029), 90% to *P*. *fluorescens* B52 lipase (P41773), 77% to *Pseudomonas* sp. MIS38 lipase (2Z8X), and 65% to *Serratia marcescens* lipase (LipA; 2QUA), respectively. The results suggested that LipB is a new lipase that may possess some unique properties. As shown in Figure 1, phylogenetic tree analysis confirmed that LipB was classified as bacterial lipase subfamily I.3. Additionally, the further multiple sequence alignment revealed that three active site residues (Ser207, Asp255, and His313) form a catalytic triad, and the GXSXG motif conserved in most microbial lipases was identified near Ser207 (Figure 2). The 3D structure of the homology model showed that one calcium ion was bound and there were four repeats of GGxGxDxUx (U: hydrophobic residue) in the sequence of LipB, termed the repeat in toxin (RTX) motif. These RTX domains bound calcium ions and formed a β-roll motif. The N-terminal domain of LipB contained the canonical hydrolase fold with two lids, and the only β-roll sandwich structure at the C-terminal domain of LipB was required for secretion and correct folding of the lipase (Figure 3A).

### 2.2. Expression and Purification of LipB

*B. subtilis* is a common prokaryotic expression host and has been successfully used to express a variety of heterologous proteins. Previously, many lipases, such as T1.2RQ [25], LipBP [26], PVL [27], and LipA [3], were successfully secreted during expression in *B. subtilis*. To study the characteristics of LipB, *B. subtilis* was selected to express the *lipB* gene. Online predictive analysis found that the *lipB* gene contains no signal sequence, so the full-length *lipB* gene sequence (approximately 1.3 kb) was synthesized after codon optimization and then expressed in *B. subtilis* WB800N via the expression vector pHT43. By inducing fermentation with β-D-1-thiogalactopyranoside (IPTG), the supernatant of fermentation was collected and detected by sodium dodecyl sulfate-polyacrylamide gel electrophoresis (SDS-PAGE) (Figure S1A). After purification (Figure S1B), dialysis, and concentration, the recombinant LipB was further analyzed by SDS-PAGE. As presented in Figure 3B, a protein band with a molecular weight approximate to the theoretical value of LipB (calculated MW = 50.0 kDa) was obtained and analyzed by mass spectrometry after purification, with the results (Figure S2) suggesting that LipB was successfully expressed in *B. subtilis*. In general, a high level of yield facilitates large-scale protein preparation. To this end, the purification fold of LipB was calculated. As shown in Table 1, a purification fold of 43.92 with 10.45% recovery was achieved. Previously, *Pseudomonas* sp. AMS8 lipase was purified 9.7-fold with 23.0% recovery via affinity chromatography and gel filtration [28]. Yang et al. [29] reported a 48.6-fold purification and 6.55% yield for *Pseudomonas* sp. R0-14 lipase through affinity chromatography. Thus, the purification of LipB in this study was at a moderate level.

### 2.3. Substrate Specificity of Recombinant LipB

**Figure 4:**
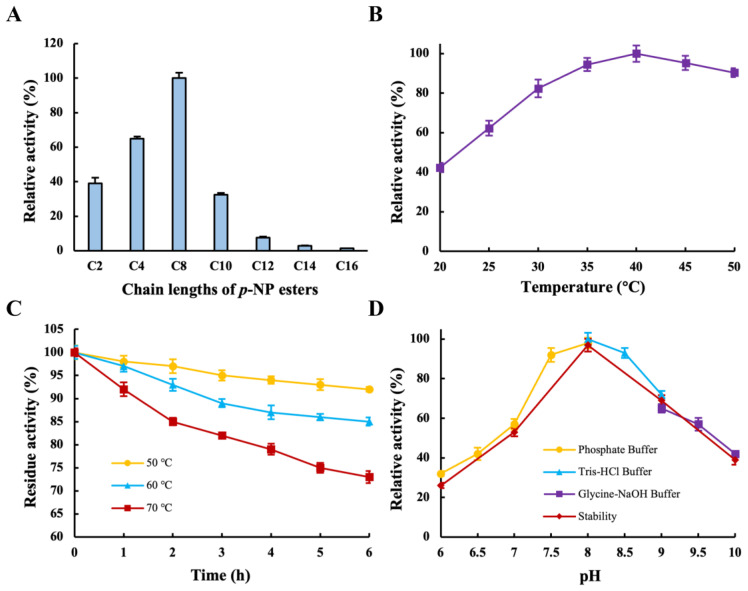
Characterization of recombinant LipB. (**A**) Substrate specificity for recombinant LipB toward various p-NP esters (C2–C16); numbers indicate carbon chain length. (**B**) Effects of temperature on recombinant LipB (20–50 °C). (**C**) Thermal stability of recombinant LipB (50 °C, 60 °C, and 70 °C). (**D**) Effects of pH on the activity and pH stability of recombinant LipB (6.0–10.0).

To investigate the substrate specificity of the recombinant LipB, the hydrolytic activity of several substrates with different carbon lengths was measured. As shown in Figure 4A, the recombinant LipB was capable of hydrolyzing triglycerides with various acyl chains (C2–C16). Among them, the optimum activity was observed when the substrate was C8, while very low activities were observed with longer acyl chains (C14–C16). In fact, the optimal hydrolase activity of the lipase from *P. fluorescens* JCM5963 was on a C8 substrate [6], whereas the lipase from *Burkholderia cepacia* RQ3 favored C14 [30]. Obviously, the substrate specificity of LipB was in accordance with most *Pseudomonas* lipases that preferred short- or medium-chain substrates, unlike the *Burkholderia* lipases, which had a higher activity towards longer-chain fatty acid esters [30]. Additionally, the majority of PUFAs in fish or algal oils have longer carbon chains, making LipB an excellent candidate for its enrichment of PUFAs.

Generally, the Michaelis–Menten constants (*K*_m_) of different bacterial lipases are not definite values and usually vary between 0.0064 mM and 16.58 mM [8]. A lower *K*_m_ value indicates a greater affinity for the enzyme for the substrate, while a higher maximum velocity (*V*_max_) value reflects a faster reaction rate [8]. To investigate the catalytic capacity of LipB, these two parameters were calculated (Figure S3). The results showed that *K*_m_ and *V*_max_ of the recombinant LipB for C8 were 0.81 mM and 76.1 μmol min^−1^ mg^−1^, respectively. The *K*_m_ of the recombinant LipB was slightly higher than *Pseudomonas* sp. AMS8 lipase (0.73) [28] but much lower than the reported lipases from *P. fluorescens* (1.63) [31] and *Burkholderia multivorans* V2 (1.56) [32], which indicated that LipB had a higher affinity for the substrate.

### 2.4. Effects of Temperature and pH on Activity and Stability of Recombinant LipB

As is known, the optimum temperature of lipase is a very important factor affecting its industrial application, and it varies for different sources of lipases. For instance, the optimal temperature of cold-adapted lipase from *Psychrobacter* sp. S1B [33] and *Pseudomonas* sp. AMS8 [28] was 30 °C, while the maximal activity of an esterase from marine environmental genomic DNA libraries emerged at 60 °C [34] and the optimum temperature of *Geobacillus stearothermophilus* FMR12 lipase reached 70 °C [35]. Consequently, the optimal temperature and thermal stability of the recombinant LipB were determined. As shown in Figure 4B, the recombinant LipB showed maximal activity at 40 °C, and the enzyme activity remained above 60% in the range of 25 °C to 50 °C. The optimal temperature of the recombinant LipB was similar to that of lipases from *B. cepacia* RQ3 [30]. According to the findings, recombinant LipB had a slightly lower optimal temperature and showed good activity across a wide temperature range. The application of lipase at lower optimal temperatures in the industry has many advantages, as it saves production costs through reduced energy consumption and increases revenue by decreasing undesired reactions and volatilization losses.

The thermostability of the recombinant LipB was tested by the preheated water bath method. As shown in Figure 4C, recombinant LipB maintained high stability after incubation at 50 °C and 60 °C for 6 h; residual activity dropped slightly and retained 73% of the initial activity after incubation at 70 °C for 6 h, which indicated that the recombinant LipB was a thermostable lipase. Gao et al. reported that the activity of *Stenotrophomonas maltophilia* OUC_Est10 lipase was preserved at less than 60% with incubation at 65 °C for 6 h [36]. The lipase from *Bacillus licheniformis* retained only 40% of its original activity after 6 h of incubation at 60 °C [37]. LipB had better thermal stability compared to the two lipases discussed above. Lipases with different optimum temperatures and outstanding thermal stability provide a variety of choices for usage in different industrial scenarios. With respect to enzymes used in detergent, it is necessary to maintain high activity and stability over a wide temperature range [8].

The influence of pH on lipase activity is mainly imputed to disrupting the intermolecular bonds of the proteins and thus changing the molecular conformation. The pH profile of recombinant LipB was studied. As shown in Figure 4D, with rising pH, the recombinant LipB activity increased and eventually dropped in the range of 6.0–10.0, and the optimum activity was obtained at pH 8.0. With regard to pH stability, the recombinant LipB was stable with a pH ranging from 7.0 to 9.0, retaining more than 55% residual activity after being incubated at 40 °C for 6 h. The optimal pH for the lipase from *Pseudomonas* sp. YY31 [38], *Clostridium* sp. CAG:413 [39], and *Pseudomonas* sp. TK-3 [40] was 8.0. *Bacillus manliponensis* lipase [39], *Pseudomonas* sp. PF16 lipase [41], and *P. fluorescens* 26-2 lipase [42] displayed the maximum activity at pH 9.0. It can be seen that LipB exhibited a similar optimal pH. In general, most microbial lipases showed optimum activity in the neutral pH range [43]. The pH property experiment suggested that the recombinant LipB was also an alkaline lipase, and the stability implies its potential in many industrial scenarios, such as the synthesis of biodiesel and enrichment of PUFAs.

### 2.5. Effects of Metal Ions, Compounds with Known Inhibitory Effect, and Detergents on Activity of Recombinant LipB

In industrial applications, reaction mixtures often contained a variety of chemicals that affected lipase activity to a greater extent. Three additives were employed to test their effects on the recombinant LipB activity. As seen in Table 2, in the presence of Na^+^, Ca^2+^, Mg^2+^, and Ba^2+^, the enzyme activity of the recombinant LipB was significantly stimulated. Especially Ca^2+^ and Mg^2+^ increased the activity of the recombinant LipB, reaching 201.53% and 182.47%, respectively, compared to the control. The results were consistent with previous studies that found Ca^2+^ and Mg^2+^ to be positive factors for lipases from *Pseudomonas stutzeri* ZS04 [44] and *Serratia* sp. W3 [45]. This activation can be attributed to the alteration of lipase conformation after binding metal ions and producing salt bridges so as to improve its stability and enhance its catalytic activity [35]. Furthermore, after incubation with K^+^, Co^2+^, Sr^2+^, and Ni^2+^, the recombinant LipB could maintain 78.76–90.18% of its initial activity. However, Cu^2+^, Zn^2+^, and Mn^2+^ significantly inhibited the activity of lipases at high concentrations. The lipase from *Pseudomonas* sp. AMS8 was confirmed to have similar properties [28].

Table 3 presents the effects of compounds with known inhibitory effects and detergents on the recombinant LipB activity. The activity of recombinant LipB was increased to 107.62% and 113.95% after treatment with Triton X-100 at concentrations of 0.1% and 1%, respectively. 0.1% Tween-20 augmented the lipase activity to 105.34%, while further increasing the concentration to 1% would inhibit the lipase activity (92.55%). Former studies also reported similar results. For example, lipases from *P. stutzeri* ZS04 [44] and *B. ubonensis* SL-4 [7] were also stimulated by Tween-20 and Triton X-100. However, the activity of recombinant LipB was significantly decreased after treatment with EDTA, PMSF, DTT, and CTAB, and the effect became more obvious with the increase in concentration. Theoretically, PMSF would completely inhibit a serine hydrolase such as LipB. However, as reported in the previous literature [32,37,46], as the serine hydrolase based on the GXSXG conserved region, different degrees of inhibition of those lipases activities by PMSF were demonstrated, but none of them were completely inactivated. This suggested that PMSF does have an inhibitory effect on serine hydrolase activity, but complete inactivation may also be related to the source of the enzyme, the concentration of the additive, and the incubation time. The highest decrease emerged in the CTAB group, remaining with 61.02% residual activity at a concentration of 1%. In contrast, *Pseudomonas punonensis* lipase activity was enhanced by EDTA [47], and Moayad et al. [48] reported that CTAB and SDS had a promotion effect on the lipase activity as well. Those diverse effects of the same additive on different lipases confirmed the need to study the effect of the additive on lipase activity before its industrial application.

### 2.6. Effects of Organic Solvents in Aqueous Mixtures on Activity of Recombinant LipB

Generally, organic solvents in a reaction medium improve the dissolution of many reactants and minimize the reaction viscosity, which lessens enzyme inhibition and increases conversion efficiency [49]. The effects of organic solvents in aqueous mixtures on recombinant LipB activity were investigated. Table 4 shows that the recombinant LipB was stable in all organic solvents selected in this study. After the treatment with organic solvents with a concentration of 10%, the recombinant LipB maintained more than 83.04% residual activity compared with the control, and two of the treatment groups showed activation. Lipase activity was inhibited in all treatment groups when the concentration reached 30%, and the activities remained at 45.79–95.74% of their original activity. In addition, the recombinant LipB showed moderate tolerance to polar organic solvents such as acetone, methanol, ethanol, glycerol, isopropanol, and acetonitrile. Acetone with a concentration of 10% strongly increased the recombinant LipB activity to 133.18%, and acetonitrile slightly reduced the residual activity to 97.63%, but the effects declined when the concentration of the above-mentioned polar organic solvents was increased to 30%. Especially for 30% ethanol, the residual activity was only 45.79% of its original activity. The stability and activation could be attributed to the flexible open conformation of lipases maintained by the interaction of the organic solvents with hydrophobic amino acid residues in the lid, which facilitates the reaction substrate to enter the active site [37]. The other reason may be due to the hydrophobic solvents assisting lipase to retain its natural structure and reduce the side reaction through decreasing the redistribution of water molecules present on the hydration shell of the lipase [50].

In general, lipases show poor stability when incubated with hydrophilic organic solvents, and this instability may be due to the hydrophilic solvents stripping the key-bound water from the enzyme molecules [51]. However, many lipases with some polar solvent tolerances have been reported. *Serratia* sp. W3 lipase maintained 77% of the initial enzyme activity by the treatment of acetone at a 10% concentration [45]. The lipase from *P. fluorescens* JCM5963 retained 65% activity when incubated with chloroform at a 30% concentration [6]. The lipase from *P. fluorescens* KE38 preserved less than 20% of its original activity compared to the control incubated with isopropanol at a concentration of 30% [52]. In comparison with the above three lipases, the recombinant LipB showed much better tolerance when incubated in the same organic solvents and maintained 133.18%, 65.24%, and 76.45% of the initial activity, respectively. Thus, the recombinant LipB displayed considerable stability during incubation with hydrophilic and hydrophobic organic solvents.

### 2.7. Enrichment of Polyunsaturated Fatty Acids with Recombinant LipB

**Figure 5:**
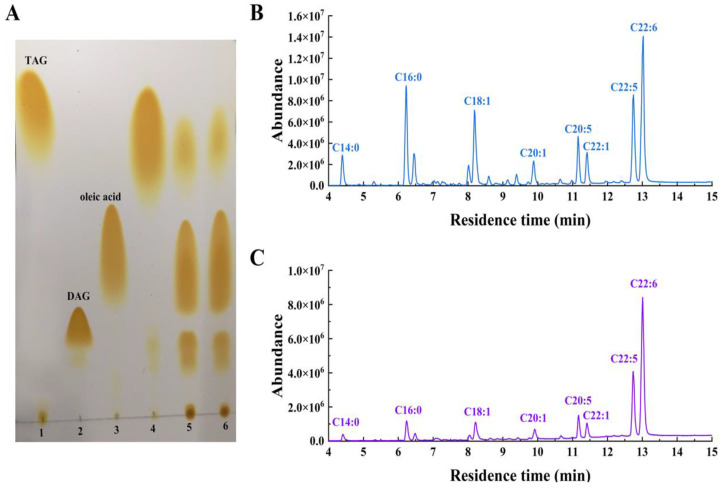
Hydrolysis of fish oil by recombinant LipB. (**A**) TLC analysis of fish oil. Lanes 1–3, standards. Lane 4, raw fish oil. Lanes 5–6, fish oil hydrolyzed for 12 and 24 h, respectively. (**B**) Analysis of the raw fish oil by GC-MS. (**C**) Analysis of fish oil after hydrolyzing for 24 h by GC-MS.

**Figure 6:**
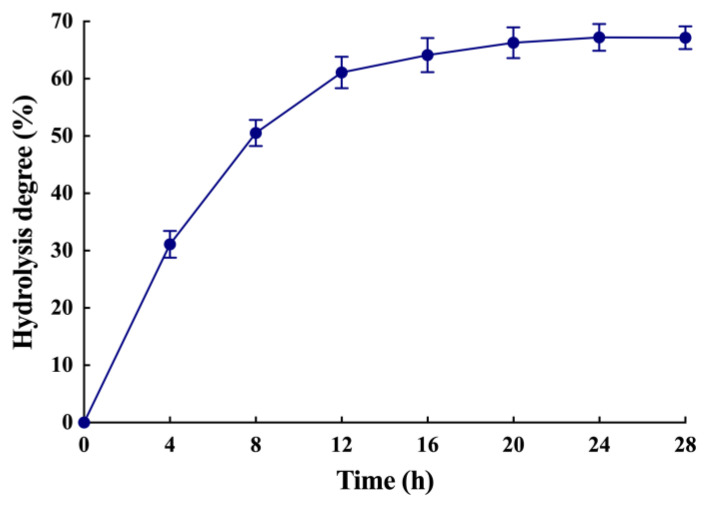
The degree of hydrolysis of fish oil. Fish oil was hydrolyzed by the recombinant LipB, and the degree of hydrolysis was measured by sampling every 4 h.

The recombinant LipB was shown to be a thermally stable and organic solvent-tolerant alkaline lipase that preferred to hydrolyze triglycerides with short and medium acyl chains. These characteristics indicated that LipB might be suited for the enrichment of PUFAs in fish oil. To prove it, the recombinant LipB was applied to fish oil hydrolysis. Firstly, the FFA profile of raw fish oil was measured by gas chromatography (GC) and mass spectrometry (MS). As presented in Figure 5B and Table 5, the raw fish oil contained 4.16% C14:0 (tetradecanoic acid), 13.22% C16:0 (hexadecanoic acid), 12.50% C18:1 (octadecenoic acid), 3.72% C20:1 (eicosenoic acid), 5.02% C20:5 (EPA), 4.51% C22:1 (docosenoic acid), 13.71% C22:5 (DPA), and 24.43% C22:6 (DHA). Total n-3 PUFAs, including EPA, DPA, and DHA, accounted for 43.16%, which indicated that fish oil is an ideal substrate for the enrichment of PUFAs. After a 24-h treatment with the recombinant LipB, fish oil was analyzed by thin-layer chromatography (TLC) and GC-MS. As shown in Figure 6, the degree of hydrolysis of fish oil gradually increased as the hydrolysis reaction proceeded, reaching 67.17% at 24 h. As shown in Figure 5A, fish oil was hydrolyzed efficiently, and the hydrolytic products, including triacylglycerols, diglycerides, monoglycerides, and FFAs, were completely separated. The GC-MS (Figure 5C and Table 5) analysis indicated that all fatty acids in fish oil were hydrolyzed to FFAs in varying degrees, but the SFAs and MUFAs were released prior to the PUFAs from the glycerin skeleton. The product contained 72.18% of n-3 PUFAs, including 5.75% EPA (66.73% recovery), 19.57% DPA (67.32% recovery), and 46.86% DHA (70.15% recovery), which were dramatically enriched and much higher than the amounts in crude fish oil.

PUFAs, especially EPA and DHA, possess more than one carbon double bond, which results in steric hindrances such as fatty acid bending and further proximity of the terminally located methyl groups to the ester bond among the glycerol backbone. More carbon double bonds will make this hindrance effect more serious [53]. As reported in some literature [12,54], lipases thus possess a higher docker interactive energy for PUFAs compared to SFAs and MUFAs. With the steric hindrance due to the molecular conformation of the PUFAs, the ester bonds formed by the glycerol backbone and the PUFAs are difficult to enter inside the lipase molecule and react with the active site, which further favors lipases such as LipB in the enrichment of PUFAs [55].

Previously, several lipases had also been applied to enrich PUFAs. The lipase from *Streptomyces violascens* increased the contents of DHA and EPA to 41.84% in codfish oil after hydrolyzing for 36 h [56]. Hydrolysis of flaxseed oil by *Actinomadura sediminis* lipase enhances PUFAs up to 50% [57]. Bovine pancreatic lipase was immobilized and confirmed to be potently enriched for PUFAs; the content was increased from 45.1% to 67.7% in fish oil after reacting for 12 h [58]. Compared to these lipases, recombinant LipB exhibited excellent enrichment ability for PUFAs.

### 2.8. Commercial Prospects for LipB

The excellent properties and applications of lipase determine its commercial prospects. As shown in Table 6, the optimum temperature and pH ranged from 30 to 50 °C and 7.0 to 9.0 for the listed lipases, where LipB exhibited similar properties (40 °C, 8.0). For temperature stability, LipB showed some superiority compared to the remaining five lipases. With respect to the two commercial Amano lipases, they had lost more than half of their enzyme activity after 1 and 4 h of incubation at 60 °C, respectively, while LipB maintained 85% of its lipase activity after 6 h of incubation. All listed lipases were applied for the enrichment of PUFAs, but the final degree of enrichment was different. LipB hydrolysis of fish oil for 24 h increased the PUFA content to 72.18%, indicating that LipB had the best enrichment capacity compared to the other five lipases. In contrast, the two commercial lipases showed a preference for SFAs in the early stage of hydrolysis (3 h), while PUFAs were released from the glycerol backbone indiscriminately as the hydrolysis reaction progressed. The temperature stability, organic solvent tolerance, and PUFA enrichment ability indicate that LipB possesses a certain potential for industrial applications and commercial prospects.

## 3. Materials and Methods

### 3.1. Strains, Plasmids, and Materials

The plasmids pUC57 (Takara, Otsu, Japan) and pHT43 (MoBiTec, Göttingen, Germany) were employed to construct the cloning and expression vectors, respectively. *Escherichia coli* TOP10 (Novagen, Darmstadt, Germany) was utilized as the host strain for plasmid amplification with ampicillin (100 μg/mL). *B. subtilis* WB800N (MoBiTec) was adopted as a heterologous protein expression strain with chloramphenicol (5 μg/mL). All strains were grown in LB medium and incubated at 37 °C. DNA sequencing was undertaken by Tsingke Biotechnology Co., Ltd. (Beijing, China). Restriction enzymes cutting off clones and expression plasmids were obtained from Thermo Fisher (Waltham, MA, USA). T4 DNA ligase ligating the target gene fragment to the enzymatically cleaved plasmid was commercially obtained from Takara. Fish oil was bought from Guangzhou Shengxuan Food Additives Co., Ltd. (Guangzhou, China) Substrate *p*-nitrophenyl (*p*-NP) esters for lipase activity determination and standards were bought from Sigma-Aldrich (St. Louis, MI, USA). All other chemicals used were of analytical grade and commercially available from Sinopharm Chemical Reagent Co., Ltd. (Shanghai, China).

### 3.2. Gene Synthesis and Sequence Analysis

The nucleotide and amino acid sequences of *lipB* were obtained from the NCBI with the accession numbers PFLU_3141 and CAI2797360, respectively. The analysis of the nucleotide sequence of *lipB* was conducted by SignalP 5.0 (http://www.cbs.dtu.dk/services/SignalP, accessed on 11 March 2022). The codon-optimized *lipB* gene with a carboxyl-terminal eight-histidine (his)-tag was synthesized and cloned into plasmid pUC57, which was completed by Wuhan GeneCreate Biological Engineering Co., Ltd. (Wuhan, China). Protein sequence similarity was analyzed using BLASTP (http://www.ncbi.nlm.nih.gov/BLAST, accessed on 8 October 2022). Multiple sequence alignment was completed through ClustalW 2.0 and ESPript 3.0 (http://espript.ibcp.fr/ESPript/ESPript, accessed on 15 October 2022). The phylogenetic tree was accomplished with MEGA 11.0, where the method used and its parameters were neighbor-joining and 1000 bootstrap replicates. The 3D structure was conducted by SWISS-MODEL (http://swissmodel.expasy.org) through homology modeling, in which the template was chosen for the lipase from *Pseudomonas* sp. MIS38. Its specific crystal structure information was available in the PDB (https://www.rcsb.org) database with the accession number 2z8x.

### 3.3. Expression and Purification

After digesting with *Bam*HI and *Aat*II, the *lipB* gene was subcloned into plasmid pHT43 and then transformed into *B. subtilis* WB800N by chemical method [63]. The recombinant strains were cultured overnight in 5 mL of LB broth and then diluted 1:100 into 500 mL shake flasks containing 50 mL of LB fresh medium and incubated in a shaker set at 200 rpm at 37 °C. When the optical density of cultures at 600 nm reached 0.7–0.8, 0.1 mM IPTG was added to the medium to induce LipB expression. After fermentation at 37 °C for 18 h, 750 mL cultures were centrifuged twice for the purpose of removing bacteria cells and insoluble impurities, where centrifugation parameters were set at 15 min and 4 °C at 8000× *g* and 12,000× *g*, respectively. After being concentrated with a 10 kDa ultrafiltration tube, the obtained supernatant was loaded onto a Ni-NTA affinity chromatography column (GE Healthcare, Chicago, IL, USA) that had been pre-equilibrated with 3 times the column volume of Tris-HCl buffer (50 mM, pH 8.0). Sequential rinsing of the packing with 10 times the column volume of Tris-HCl buffers containing 0, 30, 60, 100, and 200 mM of imidazole, respectively. The eluate was collected, dialyzed in 100 times its volume of Tris-HCl buffer, and finally concentrated using a 10 kDa ultrafiltration tube. Subsequently, the purity of the recombinant LipB was analyzed by SDS-PAGE, and the protein band was analyzed using mass spectrometry performed by Yanxing Biotech Co., Ltd. (Wuhan, China). The ratio of specific activity in purified LipB and supernatant was defined as the purification fold, while the percentage of total enzyme activity is given as the recovery. Protein concentration was determined by the Bradford method using BSA as a standard [64].

### 3.4. Biochemical Characterization

Determination of lipase hydrolytic activity using standard methods, with *p*-NP ester used as substrate [65]. Briefly, the reaction mixture consisted of 940 μL of Tris-HCl buffer (50 mM, pH 8.0), 40 μL of ethanol, 10 μL of *p*-NP ester (100 mM, dissolved in acetonitrile), and 10 μL of Tris-HCl (control) or diluted enzyme solution. After incubation at 40 °C for 5 min, the resulting mixtures were measured for absorbance at 410 nm. All experiments were conducted in triplicate. One unit of lipase activity (U) was defined as the amount of enzyme that released 1 μmol of *p*-NP per minute under the measurement conditions.

For the biochemical characterization assay, purified lipase was used with a volume of 10 µL and an enzyme activity of 64.1 U/mL. The group with the highest enzyme activity, or the non-treated group, was taken as the control (100%). Different *p*-NP esters (*p*-NP acetate (C2), *p*-NP butyrate (C4), *p*-NP caprylate (C8), *p*-NP decanoate (C10), *p*-NP laurate (C12), *p*-NP myristate (C14), and *p*-NP palmitate (C16)) were used as substrates in the determination of lipase activity, and the variation in the carbon chain length of these esters resulted in differences in the final enzyme activity, which was the way to determine the substrate specificity of lipase. The effects of temperature on recombinant LipB activity were investigated in Tris-HCl buffer (50 mM, pH 8.0). Specifically, 20 °C, 25 °C, 30 °C, 35 °C, 40 °C, 45 °C, and 50 °C were chosen to test the optimum temperature of recombinant LipB. Following this, 50 °C, 60 °C, and 70 °C were set to measure the thermal stability of recombinant LipB. The impacts of pH on the recombinant LipB activity were examined under pH 6.0, 6.5, 7.0, 7.5, 8.0, 8.5, 9.0, 9.5, and 10.0, respectively. The pH stability of the recombinant LipB was analyzed at pH 6.0, 7.0, 8.0, 9.0, and 10.0, respectively. Phosphate buffer (pH 6.0–8.0), Tris-HCl buffer (pH 8.0–9.0), and Glycine-NaOH buffer (pH 9.0–10.0) provided an acidic or alkaline environment in the corresponding interval. Two parameters (*K*m and *V*max) of recombinant LipB were calculated at optimum enzyme assay conditions with different *p*-NP C8 concentrations (0.01–2.0 mM) by the Lineweaver–Burk plot.

Metal ions (K^+^, Na^+^, Ca^2+^, Mg^2+^, Ba^2+^, Cu^2+^, Zn^2+^, Mn^2+^, Ni^2+^, Sr^2+^, Co^2+^, and Fe^3+^), compounds with known inhibitory effects (EDTA, PMSF, DTT, β-ME, and H_2_O_2_), detergents (Tween-20, Tween-80, Triton X-100, SDS, and CTAB), and organic solvents (Glycerol, DMSO, Methanol, *tert*-Butanol, Ethanol, Acetone, Acetonitrile, Isopropanol, Chloroform, Cyclohexane, and n-Hexane) were used as additives and prepared in Tris-HCl buffer (50 mM, pH 8.0) to examine their effects on the recombinant LipB activity. An amount of 10 µL of purified LipB was incubated in 940 µL of the above solution, followed by the addition of ethanol and *p*-NP ester, and then the residual enzyme activity was determined according to standard methods. The recombinant LipB was pre-incubated with different metal ions and compounds with known inhibitory effects at two concentrations (1 mM and 10 mM) and incubated at 40 °C for 30 min. The assay for the effects of detergents on the recombinant LipB activity was performed under the same conditions except for the concentration (0.1% and 1% (*w*/*v*)). Compared to the above assay, there are two differences in the process of testing the effects of organic solvents in aqueous mixtures on the recombinant LipB activity: the concentrations chosen were 10% and 30% (*w*/*v*) and the treatment was carried out at 150 rpm for 2 h. The resultant values for the effect of additives on enzyme activity were presented in the form of means ± standard deviations.

### 3.5. Treatment of Fish Oil by Recombinant LipB

#### 3.5.1. Hydrolysis Reaction

The hydrolysis reaction system consisted of 2 mL of Tris-HCl buffer (50 mM, pH 8.0), 1 g of fish oil, and 2 mL of purified recombinant LipB dissolved in Tris-HCl buffer with a concentration of 2 mg/mL. The mixture was sealed after 1 min of nitrogen blowing and then shaken at 200 rpm at a temperature of 40 °C. Samples were taken and tested at regular intervals. Reaction termination was achieved by placing the reaction mixture in a 100 °C water bath for 10 min.

#### 3.5.2. Test Sample Preparation

The reaction mixture was dissolved using 50 mL of n-hexane, followed by the addition of 20 mL of KOH ethanol solution (0.5 M) and vigorous shaking. The upper layer containing the glycerides was washed 3 times with pure water (20 mL), followed by rotary evaporation (50 r/min, 55 °C) for 20 min, and weighed. The preparation of the oil phase for methyl esterification prior to GC-MS detection is referred to in the previous description with some modifications [66]. Briefly, a 50 mg oil sample was accurately weighed and dissolved in 25 mL of n-hexane, to which 2 mL of solution was added 200 µL of KOH methanol solution (4 M), followed by vortex shaking for 2 min, and the upper layer was taken after the solution was left for 10 min.

#### 3.5.3. Hydrolysis Product Detection

The raw fish oil and the oil phase of hydrolyzed mixtures were assayed by TLC. Hexane, diethyl ether, and acetic acid were mixed at a volume ratio of 85:15:1 and then developed for the different components of the sample. The methyl esters prepared from the raw fish oil and hydrolyzed mixtures were identified and quantified with GC-MS (Agilent, USA) with flame ionization detection (FID) equipped with a capillary column (DB-WAX, 30 m × 250 µm × 0.25 µm). The temperature of the injector was 250 °C and the connector temperature was set at 260 °C. The oven temperature was 150 °C, which was then heated to 250 °C at a rate of 10 °C/min and maintained for 10 min. For the MS program, the temperatures of the MS quad and MS source were set at 150 °C and 230 °C, respectively. The solvent delay time was set to 3 min, and the rate of He flow was 1 mL/min. Identification of fatty acid methyl esters based on the retention time of the standards in GC-MS analysis and quantification using the external standard method.

The values of acid and saponification were detected according to the methods in the Chinese National Standards GB/T 5530-2005 and GB/T 5334-2008. The degree of hydrolysis was calculated according to the following formula:

Degree of hydrolysis (%) = [(AVt − AV)/(SV − AV)] × 100

where AVt is the acid value of the hydrolysis product, AV is the acid value of raw fish oil, and SV is the saponification value of raw fish oil.

The recovery yield of n-3 PUFAs (EPA, DPA, and DHA) was calculated according to the following formula:

Recovery yield (%) = [(Ct × Mt)/(C × M)] × 100

where Ct and C are the contents of n-3 PUFAs in the hydrolyzed glyceride product and raw fish oil. Mt and M are the masses of the hydrolyzed glyceride product and raw fish oil.

## 4. Conclusions

In this study, a new lipase, LipB from *P. fluorescens* SBW25, was expressed in *B. subtilis* WB800N. Its purified form showed excellent thermal stability, high tolerance to organic solvents, and good enrichment ability of n-3 PUFAs from fish oil. After hydrolyzing for 24 h, LipB could improve the contents of EPA, DPA, and DHA from 43.16% to 72.18%. Our research results demonstrated the great potential of LipB in the production of n-3 PUFAs in the food industry.

## Figures and Tables

**Table 1:** Purification of recombinant LipB.

Steps	Total Protein (mg)	Total Activity (U)	Specific Activity (U/mg)	Purification (fold)	Recovery (%)
Supernatant	2060	3680	1.78	1	100
Purified LipB	4.92	384.6	78.17	43.92	10.45

**Table 2:** Effects of metal ions on the activity of recombinant LipB.

Metal Ions	Relative Activity (%)	
	1 mM	10 mM
Control	100 ± 1.24	100 ± 1.06
K^+^	99.47 ± 2.16	78.76 ± 3.05 **
Na^+^	102.16 ± 2.16	111.49 ± 1,87 **
Ca^2+^	142.19 ± 1.79 **	201.53 ± 2.09 **
Mg^2+^	157.27 ± 1.18 **	182.47 ± 2.56 **
Ba^2+^	141.86 ± 1.29 **	179.45 ± 1.61 **
Cu^2+^	71.03 ± 1.80 **	31.62 ± 1.81 **
Zn^2+^	75.42 ± 2.15 **	52.04 ± 1.38 **
Mn^2+^	90.58 ± 2.59 **	52.45 ± 1.03 **
Ni^2+^	95.84 ± 1.28 *	87.53 ± 1.84 **
Sr^2+^	98.58 ± 2.27	90.18 ± 1.91 **
Co^2+^	90.67 ± 1.76 **	84.05 ± 2.18 **
Fe^3+^	89.33 ± 1.58 **	70.61 ± 1.54 **

* *p* < 0.05, ** *p* < 0.01, compared with control.

**Table 3:** Effects of compounds with known inhibitory effects and detergents on the activity of recombinant LipB.

Additives	Relative Activity (%)	Relative Activity (%)
Compounds with known inhibitory effect	1 mM	10 mM
Control	100 ± 1.94	100 ± 1.14
EDTA	82.29 ± 2.49 **	68.61 ± 2.73 **
PMSF	80.35 ± 1.94 **	67.51 ± 1.46 **
DTT	73.16 ± 2.26 **	66.85 ± 1.89 **
β-ME	86.32 ± 1.73 **	77.83 ± 1.29 **
H_2_O_2_	80.55 ± 1.13 **	73.6 ± 1.82 **
Detergents	0.1%	1%
Control	100 ± 1.41	100 ± 1.38
Tween-20	105.34 ± 1.71 **	92.55 ± 2.61 **
Tween-80	98.75 ± 2.53	87.19 ± 1.95 **
Triton X-100	107.62 ± 2.85 **	113.95 ± 1.43 **
SDS	92.46 ± 1.816 **	70.25 ± 2.27 **
CTAB	87.36 ± 1.21 **	61.02 ± 2.25 **

** *p* < 0.01, compared with control.

**Table 4:** Effects of organic solvents in aqueous mixtures on the activity of recombinant LipB.

Organic Solvents	log P	Relative Activity (%)	
		10%	30%
Control		100 ± 2.89	100 ± 1.91
Glycerol	−3.03	91.87 ± 1.23 **	90.65 ± 1.67 **
DMSO	−1.3	93.12 ± 1.09 **	86.53 ± 2.36 **
Methanol	−0.76	91.25 ± 2.02 **	56.47 ± 1.42 **
Ethanol	−0.24	83.04 ± 2.12 **	45.79 ± 1.38 **
Acetone	−0.23	133.18 ± 2.66 **	95.74 ± 1.52 **
Acetonitrile	−0.15	97.63 ± 1.44	91.18 ± 1.99 **
Isopropanol	0.1	95.26 ± 1.64 **	76.45 ± 1.52 **
*tert*-Butanol	0.84	87.58 ± 2.17 **	72.35 ± 1.98 **
Chloroform	2.0	84.51 ± 1.72 **	65.24 ± 1.17 **
Cyclohexane	3.2	92.75 ± 1.41 **	83.45 ± 1.64 **
n-Hexane	3.5	102.18 ± 2.86	92.57 ± 1.52 **

** *p* < 0.01, compared with control.

**Table 5:** The fatty acid profile (%) of raw fish oil and hydrolysis products after treatment by the recombinant LipB.

Fatty Acids	Raw Fish Oil	Hydrolysis Product
C14:0	4.16 ± 0.07	1.91 ± 0.03
C16:0	13.22 ± 0.25	5.89 ± 0.07
C16:1	4.95 ± 0.14	2.22 ± 0.05
C18:0	2.75 ± 0.06	1.46 ± 0.11
C18:1	12.50 ± 0.19	6.70 ± 0.24
C20:1	3.72 ± 0.13	3.20 ± 0.12
C20:5	5.02 ± 0.08	5.75 ± 0.08
C22:1	4.51 ± 0.16	5.11 ± 0.18
C22:5	13.71 ± 0.22	19.57 ± 0.25
C22:6	24.43 ± 0.25	46.86 ± 0.19
Other FAs	11.03 ± 0.19	1.33 ± 0.05
Total n-3 PUFAs	43.16 ± 0.28	72.18 ± 0.52

**Table 6:** Comparison of some lipases and LipB in particular properties and applications.

Lipases	Optimum Temperature (°C)	Temperature Stability (°C, h, and %)	Optimum pH	Enrichment of PUFAs (%)	References
*P. fluorescens* SBW25 lipase	40	60, 6, 85	8.0	EPA + DPA + DHA 43.16 to 72.18	This study
*P. fluorescens* MTCC 2421 lipase	40	50, 1, 40	8.0	EPA 17.8 to 35.28	[59]
*Burkholderia gladioli* BRM58833 lipase	50	60, 1.2, 50	9.0	DHA 0 to 19.5	[60]
*Steptomyces violascens* ATCC 27,968 lipase	30	60, 6, 58	9.0	EPA + DHA 14.95 to 41.84	[56]
Amano *Burkholderia cepacia* lipase	40	60, 1, 40	7.0	EPA + DHA 28.34 to 11.06	[61,62]
Amano *P. fluorescens* lipase	45	60, 4, 50	8.0	EPA + DHA 28.34 to 10.73	[31,62]

## Data Availability

Not applicable.

## Supplementary Materials

The supporting information can be downloaded at: https://www.mdpi.com/article/10.3390/ijms24108924/s1.
