# Supplementary material for: Characterization of a New Thermostable and Organic Solution-Tolerant Lipase from Pseudomonas fluorescens and Its Application in the Enrichment of Polyunsaturated Fatty Acids
Source: Int J Mol Sci. 2023 May 18;24(10):8924. doi: 10.3390/ijms24108924 (PMC10219423; doi:10.3390/ijms24108924)
Supplement: Supplementary file 1 [file ijms-24-08924-s001.zip › ijms-2349635-supplementary.pdf]

SUPPLEMENTARY MATERIAL

Characterization of a New Thermostable and Organic Solution-Tolerant Lipase from *Pseudomonas fluorescens* and Its Application in the Enrichment of Polyunsaturated Fatty Acids

Zhiming Hu †, Liangcheng Jiao †, Xiaoman Xie, Li Xu, Jinyong Yan, Min Yang * and Yunjun Yan *

Key Laboratory of Molecular Biophysics of the Ministry of Education, College of Life Science and Technology, Huazhong University of Science and Technology, Wuhan 430074, China

* Correspondence: ymyangmin@hust.edu.cn (M.Y.); yanyunjun@hust.edu.cn (Y.Y.)

† These authors contributed equally to this work.

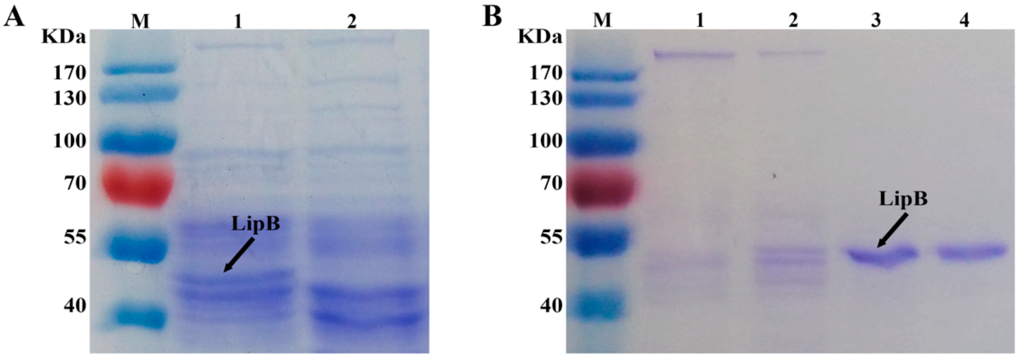

Figure S1. SDS-PAGE analysis of expression and purification recombinant LipB. (A) SDS-PAGE analysis of crude enzyme. Lane M, protein mass marker (Fermentas, SM0671). Lane 1, crude enzyme. Lane 2, culture supernatant of *B. subtilis* without the target gene. (B) SDS-PAGE analysis of affinity chromatography. Lane M, protein mass marker (Fermentas, SM0671). Lanes 1-4, eluates with imidazole concentrations of 30, 60, 100, and 200 mM, respectively.

MGIFDYKNLG TEGSKALFAD AMAITLYTYH NLDNGFAVGY QHNGLGLGLP ATLVGALLGS TDSQGVIPGL 70

PWNPDSEKAA LDAVQKAGWT PISASTLGYG GKVDARGTFF GEKAGYTTAQ VEVLGKYDDA GKLLEIGIGF 140

RGTSGPRETL ISDSIGDLVS DLLAALGPKD YAKNYAGEAF GGLLKNVADY ASAHGLSGKD VLVSGHSLGG 210

LAVNSLADLS VNKWGGFYKD AHYVAYASPT QSAGDKVLNI GYENDPVFRA LDGSSFNLSS LGVHDKPHES 280

TTDNIVSFND HYASTLWNVL PFSIANLPTW LSHLPTGYGD GMTRIVESGF YEQMSRDATV IVANLSDPAR 350

ANTWVQDLNR NAEPHKGNTF IIGSDGDDLI QGGKGVDFIE GGKGNDTLRD NSGHNTFLFS GHFGQDRVIG 420

YQLTDKLVFK DVQGSVDYRE HGGDTVISVG GDSVTLVGVS GGLGEVVIG 469

Figure S2. Mass spectrometry analysis of LipB. Purified LipB was subjected to SDS-PAGE, and the single band obtained was analyzed by mass spectrometry, with matching peptides shown in red.

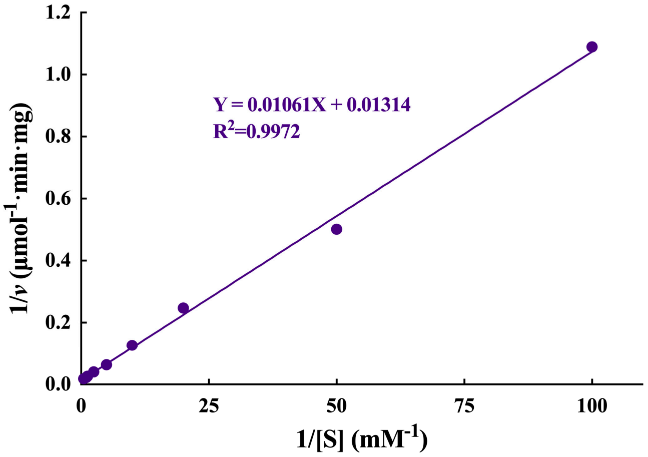

Figure S3. The Lineweaver-Burk plot of the recombinant LipB. Two parameters (*K*m and *V*max) were calculated with *p*-NP caprylate as the substrate.
